# ReCIDE: robust estimation of cell type proportions by integrating single-reference-based deconvolutions

**DOI:** 10.1093/bib/bbae422

**Published:** 2024-08-23

**Authors:** Minghan Li, Yuqing Su, Yanbo Gao, Weidong Tian

**Affiliations:** State Key Laboratory of Genetic Engineering, Department of Computational Biology, School of Life Sciences, Fudan University, 2005 Songhu Road, Yangpu District, Shanghai 200438, China; State Key Laboratory of Genetic Engineering, Department of Computational Biology, School of Life Sciences, Fudan University, 2005 Songhu Road, Yangpu District, Shanghai 200438, China; Shanghai SPH Jiaolian Pharmaceutical Technology Company, Limited, Buliding 4, 998 Ha Lei Road, Pudong District, Shanghai 201203, China; State Key Laboratory of Genetic Engineering, Department of Computational Biology, School of Life Sciences, Fudan University, 2005 Songhu Road, Yangpu District, Shanghai 200438, China; Children’s Hospital of Fudan University, 399 Wanyuan Road, Minhang District, Shanghai 201102, China; Children’s Hospital of Shandong University, 23976 Jingshi Road, Huaiyin District, Jinan, Shandong 250022, China

**Keywords:** deconvolution, rare cell types, subject-specific references, RNA-seq

## Abstract

In this study, we introduce Robust estimation of Cell type proportions by Integrating single-reference-based DEconvolutions (ReCIDE), an innovative framework for robust estimation of cell type proportions by integrating single-reference-based deconvolutions. ReCIDE outperforms existing approaches in benchmark and real datasets, particularly excelling in estimating rare cell type proportions. Through exploratory analysis on public bulk data of triple-negative breast cancer (TNBC) patients using ReCIDE, we demonstrate a significant correlation between the prognosis of TNBC patients and the proportions of both T cell and perivascular-like cell subtypes. Built upon this discovery, we develop a prognostic assessment model for TNBC patients. Our contribution presents a novel framework for enhancing deconvolution accuracy, showcasing its effectiveness in medical research.

## Introduction

Single-cell ribonucleic acid-sequencing (scRNA-seq) enables quantification of gene expression in individual cells, allowing for characterization of cellular heterogeneity within complex tissues. Despite its wide applications, scRNA-seq has been adopted to a limited extent in large-scale clinical cohort studies because of its cost [1]. Although bulk RNA-seq and microarray technologies captures averaged gene expression across heterogeneous cell types in a tissue, they have been widely applied in biomedical research because of their cost-efficiency [2]. A wealth of bulk RNA-seq and microarray cohort data are available in publicly accessible databases such as Gene Expression Omnibus (GEO) and the Cancer Genome Atlas (TCGA) [3, 4], with many complemented by valuable clinical information [5]. Utilizing cell type deconvolution, a technique that estimates cell type proportions in bulk RNA-seq and microarray data by using scRNA-seq data as a reference, these extensive cohorts of clinically informative bulk datasets can be reanalyzed, from which clinically relevant cell types can be identified to aid in the prediction of disease prognosis and a deeper understanding of disease pathogenesis [6, 7].

A typical deconvolution method would first obtain the expression profiles of cell type-specific marker genes, either from prior knowledge or derived directly from a reference of scRNA-seq data, and then employ a regression or machine learning–based method to estimate cell type proportions in bulk expression profiles. Representative methods include CIBERSORT [8], Fardeep [9], and DWLS [10]. These methods usually do not model the variations in cell-level gene expression profiles between subjects in the reference because earlier scRNA-seq studies typically included only a few subjects. With increasing throughput and decreasing cost of scRNA-seq technology, it is now not uncommon to find dozens or even hundreds of samples included in one single scRNA-seq study. Accordingly, recently developed methods start to incorporate cross-subject variations in cell-level gene expression profiles into their methodologies. These methods include MuSiC [11], Bisque [12], and BayesPrism [13]. Additionally, as multiple scRNA-seq studies become available for the same tissue of origin, methods such as SCDC-ENSEMBLE were developed to integrate the results produced by each single-cell reference set [14]. A recently developed method—interRD—further integrated multiple single-cell reference sets and prior knowledge about cell type-specific marker genes and the population-level mean proportion of each cell type in a deconvolution scheme [15].

For current deconvolution methods, a major challenge in deconvolution is the batch differences between bulk and single-cell reference data, which are likely produced by both technical and biological reasons [12]. The incorporation of cross-subject variations and the integration of multiple scRNA-seq reference sets further exacerbate the situation. To date, most deconvolution methods attempted to solve these problems within one statistical framework. However, accurately addresses all these intertwined factors with one statistical framework is not an easy task [14]. An alternative approach that can take advantage of the increasing number of available subjects and reference sets without adding extra complexity is to apply a single reference-based method, such as CIBERSORT or DWLS, to conduct deconvolution separately by using each subject’s single-cell scRNA-seq data as a reference, and then integrate the deconvolution results afterwards. Thus, here we propose a general framework called ReCIDE (Robust estimation of Cell type proportions by Integrating single-reference-based DEconvolutions), which introduces an innovative approach for robustly estimating cell type proportions by integrating single-reference-based deconvolutions. Given multiple deconvolution results produced by different scRNA-seq references, ReCIDE conducts dimension reduction and clustering to get high-confidence deconvolution results. Additionally, ReCIDE includes a module for identifying the marker gene matrix (MGM) based on the scRNA-seq references by optimizing the cosine similarity between the MGM and the bulk dataset, as a way to minimize the batch effect between the reference and target bulk datasets.

In our unbiased benchmarks, the ReCIDE-optimized deconvolution methods showed significantly improved deconvolution accuracy over the corresponding single reference-based deconvolution methods used to produce the initial deconvolution results, and ReCIDE-optimized DWLS consistently outperformed the other ReCIDE-optimized methods. Moreover, ReCIDE-optimized DWLS demonstrated superior accuracy over current multi-reference-based methods, such as MuSiC, Bisque, and BayesPrism, and multi-reference set-based methods, such as SCDC-ENSEMBLE. The primary advantage of ReCIDE–DWLS over existing methods is its outstanding performance in estimating the proportions of rare cell types (comprising less than 2% of the total cell population). This superiority is further validated through evaluations using real bulk RNA-seq and microarray datasets, where ReCIDE–DWLS successfully identified rare cell types with known clinical significance, while other competing methods failed to do so.

Finally, ReCIDE-optimized DWLS was applied to the bulk RNA-seq and microarray datasets of triple-negative breast cancer (TNBC) patients, revealing between subclasses of T cells and perivascular-like (PVL) cells, both of which are rare cell types, and the prognosis of TNBC patients, demonstrating its clinical relevance. Based on this, a prognostic evaluation model was established using the proportions of PVL cells and their subgroups, which was used to assess the prognosis of TNBC patients. ReCIDE is therefore of compelling interest for reanalyzing large cohort of clinical bulk RNA-seq data and uncovering clinically significant cell types. ReCIDE is available as an open-source R package at https://github.com/TianLab-Bioinfo/ReCIDE.

## Materials and methods

### The workflow of ReCIDE

The ReCIDE workflow comprises three primary steps: constructing and refining individual references’ cell type-specific MGMs, deconvolution using each MGM, and integrating the deconvolution outcomes.

Step 1 involves preprocessing the scRNA-seq reference matrices (counts) along with subject and cell type labels. ReCIDE first organizes the reference matrix into subject-specific ones based on subject labels. Each subject-specific matrix undergoes marker gene selection using the cosg() function from the COSG R package, initially picking 100 marker genes for each cell type [16]. Subsequently, a filtering process, as recommended by Cobos et al. [17], is applied based on the average expression fold change between the cell type with the highest and second-highest expression (SecondFC). Marker genes with SecondFC greater than 1.5 are retained. The filtered reference matrix subset, with marker genes as rows and cells as columns, is then used to build the MGM matrix via the buildSignatureMatrixUsingSeurat() function in DWLS, aiming for the highest stability following Newman et al.’s method [8]. ReCIDE further optimizes the MGM based on the correlation between reference expression profiles of cell type-specific marker genes and the target bulk expression profile. Specifically, ReCIDE sorts the marker genes of each cell type in ascending order according to their SecondFC values and then proceeds to assess each marker gene in the list. If removing a marker gene result in an improvement in the similarity score between the expression profile of the marker genes and the target bulk gene expression profile, it is removed; otherwise, it is retained. Here, the similarity score is calculated as the product of the number of marker genes for a specific cell type and the cosine similarity between the expression profiles of those marker genes and the target bulk gene expression profiles.

Step 2 involves deconvolution with individual references. ReCIDE uses each subject’s MGM obtained in Step 1 independently for deconvoluting the target bulk dataset, experimenting with FARDEEP, CIBERORT, and DWLS as deconvolution methods. After benchmark testing, the solveDampenedWLS() function of DWLS is recommended as the deconvolution kernel.

In Step 3, ReCIDE integrates and refines the predicted results from Step 2. Assuming *n* individual reference subjects, ReCIDE performs principal component analysis (PCA) dimensionality reduction on the *n* sets of deconvolution outcomes based on cell types, utilizing the fast.prcomp() function in R [18]. Subsequently, the reduced-dimensional results are clustered using Gaussian mixture models by the Mclust function from the Mclust R package [19]. After that, ReCIDE retains the largest class in the clustering result. For each cell type, the average of the estimated proportions from the largest class is considered the best estimate. Finally, ReCIDE normalizes the estimates across all cell types to ensure their sum equals 1.

### Data collection

#### Benchmark studies

For benchmark studies, various scRNA-seq datasets were employed to create pseudo-bulk data and conduct benchmark tests. The datasets used were as follows:

1. Leave-one-out test

—Pancreatic (PAN) dataset: obtained from Fasolino et al. [20] via the interactive website https://cellxgene.cziscience.com/.

—Kidney datasets: derived from Lake et al. [21], with accession number GSE183276 in the GEO database.

2. Cross-dataset test

—Prefrontal cortex (PFC) datasets: utilized snRNA-seq datasets from Xiong et al. [22] (accessed at https://www.synapse.org/) and Morabito et al. (GSE174367) [23]. Data from Xiong et al. served as the reference set, while data from Morabito et al. was used to generate pseudo-bulk data.

—Peripheral blood mononuclear cell (PBMC): two PBMC scRNA-seq datasets were downloaded from Stephenson et al. (https://covid19cellatlas.org) [24] and Perez et al. (GSE174188) [25]. Data from Perez et al. served as the reference set, while data from Stephenson et al. was used for pseudo-bulk data. Note that SciBet [26] was used to ensure consistent cell type annotations across datasets.

3. Multi-studies test

—PBMC datasets: PBMC scRNA-seq datasets from Stephenson et al. and Hao et al. (CITE-seq, GSE164378) [27] served as the reference sets, with data from Perez et al. used for pseudo-bulk data.

—Estrogen receptor-positive breast cancer (ER) datasets: included datasets from Wu et al. (GSE176078) [28], Pal et al. (GSE161529) [29], and Bassez et al. (http://biokey.lambrechtslab.org) [30]. Data from Wu et al. and Pal et al. were the reference sets, while data from Bassez et al. were used for pseudo-bulk data. SciBet ensured consistent cell type annotations across datasets.

4. High-resolution test

—Colorectal cancer (CRC) dataset: derived from Pelka et al. [31], with accession number GSE178341 in the GEO database.

—TNBC datasets: obtained from Wu et al. (GSE176078) [28].

#### Real studies

To further assess ReCIDE–DWLS under real biological conditions and explore its broader applicability, five microarray and bulk RNA-seq datasets from COVID-19, SLE, and TNBC patients were used for additional testing:

—COVID-19 dataset: bulk RNA-seq data from Matthew et al., with accession number GSE231409 in the GEO database. PBMC scRNA-seq data from Stephenson et al. [24] served as the reference set.

—SLE dataset: microarray data from Kennedy et al. [32], with accession number GSE50772 in the GEO database. PBMC scRNA-seq data from Stephenson et al. served as the reference set.

—TNBC dataset: microarray data from Jézéquel et al. [33] (GSE58812), bulk RNA-seq data from Loibl et al. [34] (GSE164458), and the TCGA (accessed at https://xenabrowser.net/). Four TNBC scRNA-seq datasets, which are from the studies of Wu et al. (GSE176078) [28], Pal et al. (GSE161529) [29], Xu et al. (GSE180286) [35], and Bassez et al (http://biokey.lambrechtslab.org) [30] were integrated as the reference set.

In this study, all datasets used in the benchmark studies were downloaded on December 15, 2023, while those used in the real studies were downloaded on December 22, 2023.

### Evaluation metrics

To comprehensively evaluate the accuracy of different deconvolution methods, we employed two metrics: root mean square error (RMSE) and Pearson correlation coefficient (PCC). RMSE quantifies the absolute error between the deconvolution results and the ground truth, providing insights into the precision of the estimation. On the other hand, PCC measures the correlation between the deconvolution outcomes and the true values, offering insights into the consistency of the predictions. RMSE was computed using the rmse() function sourced from the ModelMetrics R package [36], and PCC was determined using the cor() function from the stats R package [37].

### Construction and visualization of triple negative breast cancer single-cell reference sets

The TNBC reference atlases in this study were compiled from four sets of studies (Wu et al. (GSE176078) [28], Pal et al. (GSE161529) [29], Xu et al. (GSE180286) [35], and Bassez et al. [30]). To ensure consistency in cell type annotation, we employed the SciBet v1.0 function [26] to transfer cell type labels from the study of Wu et al. to the other three datasets, using default parameters. Visualizing the reference data involved initially removing batch effects with the IntegrateData() function from the Seurat v4.4.0 R package [27]. Subsequently, 2000 variable genes were utilized for PCA to reduce dimensionality, followed by the selection of the 30 most informative principal components (PCs) for clustering and visualization using uniform manifold approximation and projection (UMAP) [38, 39]. PCA and UMAP were executed through the RunPCA() and RunUMAP() functions from the Seurat v4.4.0 R package, respectively.

### Statistical tests

The Wilcoxon test was conducted using the stat_compare_means() function from the ggpubr R package [40, 41]. The pairwise_survdiff() and ggsurvplot() functions from the survminer R package were used for plotting Kaplan–Meier plots and log-rank tests [42]. Fisher’s test was conducted using the fisher.test() function from the stats R package [37].

### Competing methods

#### CIBERSORT

CIBERSORT was obtained from https://github.com/Moonerss/CIBERSORT and utilized following the instructions provided at https://github.com/Moonerss/CIBERSORT/blob/main/README.md. The inputs included the bulk data and MGM. MGM construction began by initially selecting 100 marker genes for each cell type using the cosg() function from the COSG R package [16]. Marker genes with a SecondFC (Second Fold Change) greater than 1.5 were retained, and the mean expression values of marker genes for each cell type were utilized to construct the MGM. All other parameters were left at their default settings.

#### DWLS

The DWLS R package v0.1.0 was obtained from https://github.com/dtsoucas/DWLS and utilized by following the tutorials provided at https://github.com/dtsoucas/DWLS/blob/master/Manual.docx. Inputs for DWLS included the bulk data, raw counts gene expression matrix of reference scRNA-seq data, and cell type labels, with the latter being annotations from the reference scRNA-seq dataset. To enhance computational efficiency, the findmarker() function from the Seurat package, invoked by the buildSignatureMatrixUsingSeurat() function in DWLS, was substituted with the cosg() function from the COSG R package [16, 27]. All other parameters were maintained at their default settings.

#### FARDEEP

The FARDEEP R package v1.0.1 was obtained from https://github.com/YuningHao/FARDEEP and utilized by following the tutorials available at https://github.com/YuningHao/FARDEEP/blob/master/Rpackage/FARDEEP/R/fardeep.R. Inputs for FARDEEP included the bulk data and the MGM, constructed in a manner similar to CIBERSORT. All other parameters were left at their default settings.

#### BayesPrism

The BayesPrism R package v2.0 was obtained from https://github.com/Danko-Lab/BayesPrism and utilized by following the tutorial available at https://github.com/Danko-Lab/BayesPrism/blob/main/tutorial_deconvolution.pdf. Inputs for BayesPrism included the bulk data, the raw counts gene expression matrix of reference scRNA-seq data, cell type labels, and cell state labels. Cell type labels were derived from annotations in the reference scRNA-seq dataset. In accordance with the benchmark paper by Tran et al. [43], we opted not to utilize the cell state labels option to ensure comparability with other methods, specifying only cell types for BayesPrism. When tumor cells were present, the key parameters in the new.prism() function were configured accordingly, while all other parameters were set to the default options of the algorithm.

#### BisqueRNA (Bisque)

The Bisque R package v1.0.5 was obtained from https://github.com/cozygene/bisque and utilized by following the guidelines provided in the tutorials available at https://github.com/cozygene/bisque/blob/master/vignettes/bisque.Rmd. Inputs for Bisque included the bulk data, the raw counts gene expression matrix of reference scRNA-seq data, cell type labels, and reference sample labels. Cell type labels were obtained from annotations in the reference scRNA-seq dataset, while reference sample labels corresponded to sample labels from the reference dataset. All other parameters were configured to the default settings of the algorithm.

#### MuSiC

The MuSiC R package v1.0.0 was obtained from https://github.com/xuranw/MuSiC and implemented by following the instructions provided in the tutorials at https://xuranw.github.io/MuSiC/articles/MuSiC.html. Inputs to MuSiC included the bulk data, the raw counts gene expression matrix of reference scRNA-seq data, cell type labels, and reference sample labels. Cell type labels were derived from annotations in the reference scRNA-seq dataset, while reference sample labels corresponded to sample labels from the reference dataset. All other parameters were left at their default settings.

#### SCDC

The SCDC R package v0.0.0.900 was obtained from https://github.com/meichendong/SCDC and utilized following the guidelines provided in the tutorials at https://meichendong.github.io/SCDC/articles/SCDC.html. Prior to deconvolution, the raw counts gene expression matrix of the reference scRNA-seq data, along with cell type labels and reference sample labels, were fed into the SCDC_qc() function for quality control (QC). Here, the cell type labels were those annotated in the reference scRNA-seq dataset, and the reference sample labels were obtained from the reference dataset. If multiple reference datasets from different studies were available, they were processed separately using the SCDC_qc() function. Given that the SCDC_qc() function is limited to handling single-cell matrices with up to 65 536 cells at most, if the number of cells in the reference dataset exceeded this limit, 60 000 cells were randomly selected for QC. Following completion of QC, for reference subjects from the same study, the bulk data, output from the SCDC_qc() function, along with cell type labels and reference sample labels, were input into the SCDC_prop() function for deconvolution. However, if reference subjects were from different studies, the SCDC_ENSEMBLE() function was utilized for deconvolution. In this case, bulk data, output from the SCDC_qc() function, cell type labels, and reference sample labels were provided as inputs. All other parameters were left at their default settings.

#### inteRD

The inteRD R package v0.1.1 was obtained from https://github.com/chencxxy28/InteRD and utilized according to the guidelines provided in the tutorials at https://chencxxy28.github.io/InteRD/articles/NAME-OF-VIGNETTE.html. To execute inteRD, the input includes bulk data, the output generated by the SCDC_ENSEMBLE() function, cell type labels, and a list of cell type-specific marker genes constructed similarly to CIBERSORT. All additional parameters were left at their default settings.

## Results

### A brief description of the ReCIDE method and the evaluation of deconvolution methods

**Figure 1:**
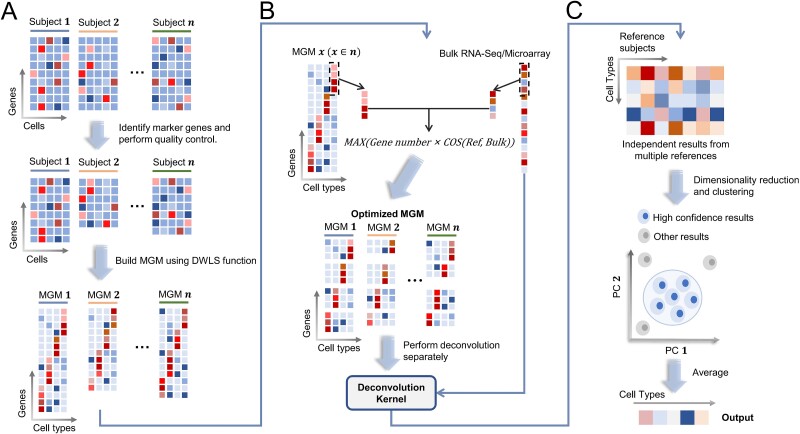
Workflow of ReCIDE. (a) Construction of subject-specific reference MGMs. (b) Optimization of subject-specific reference MGMs and separate deconvolutions on the target bulk data. (c) PCA dimensionality reduction and GMM clustering on the deconvolution results, yielding the final outcomes.

The workflow of ReCIDE comprises three primary steps: constructing and refining individual references’ cell type-specific MGMs, deconvolution using each MGM, and integrating the deconvolution outcomes (Fig. 1). For detailed information about these steps, please refer to the Materials and methods section. Here, we briefly describe these steps:

Construction and refinement of MGMs: initially, ReCIDE constructs single-cell gene expression matrices for each subject derived from a reference matrix and establishes a cell type-specific MGM following standard procedures [8, 17]. Each MGM is further optimized to maximize the similarity score between the MGM expression profile and the target expression profile.

Deconvolution: ReCIDE iteratively employs single-reference-based deconvolution approaches such as CIBERSORT, DWLS, and FARDEEP on the target bulk expression profile using each subject-specific MGM as a reference.

Integration of deconvolution outcomes: our major assumption is that if there exists a large cluster of cell type proportions, then the true set of cell type proportions is likely approximated by those within the cluster. Accordingly, for each cell type, ReCIDE computes the average of the corresponding proportions from the deconvolution results within the identified cluster and considers it the optimal estimation.

Finally, ReCIDE normalizes the estimations for all cell types to ensure their collective sum equals one. This comprehensive approach enhances the precision and robustness of cell type deconvolution, providing a refined and reliable estimation of cellular composition.

To assess the performance of ReCIDE, we devised four distinct benchmark scenarios. The first scenario, denoted as the “leave-one-out test”, involves iteratively selecting one subject from the scRNA-seq dataset as a test subject, while the remaining subjects are employed as references for deconvolving the pseudo-bulk expression profile of the test subject. In this scenario, there are minimal batch effects between the reference subjects and the target bulk data. The second scenario, termed the “cross-dataset test”, incorporates scRNA-seq reference and pseudo-bulk data sourced from different studies. This set of tests introduces more realistic batch differences in comparison to the first scenario, reflecting diverse experimental conditions. The third scenario, labeled the “multi-studies test”, combines scRNA-seq data from two distinct studies to form a reference, while pseudo-bulk data from a third study are used for testing. This design accounts for the limited number of reference subjects available from a single study and introduces batch differences across studies within the reference set. The fourth scenario, designated the “high-resolution test”, annotates scRNA-seq datasets at a finer level (>40 cell types) to specifically evaluate ReCIDE’s performance on rare cell types (constituting <2% of cells). This set of tests is also leave-one-out type, as it is difficult to guarantee the accuracy of cell type annotations across datasets at the high-resolution. For each of the aforementioned scenarios, we conducted two benchmark tests, ensuring a comprehensive evaluation of ReCIDE’s efficacy under diverse conditions.

ReCIDE implements a single-reference-based deconvolution method in its pipeline. Here, we conducted an evaluation of three best-performing single-reference-based deconvolution methods (CIBERSORT, DWLS, and FARDEEP) recommended by Cobos et al. [17] to guide the selection of the deconvolution kernel for ReCIDE. Then, we compared ReCIDE with four multi-reference-based deconvolution methods (Bisque, BayesPrism, SCDC, and MuSiC) investigated by Chu et al. [13]. InteRD is a recently published method specifically on utilizing reference datasets from multiple studies [15]. It is therefore compared with ReCIDE in the scenario of “multi-studies test”.

### Evaluation of ReCIDE’s performance on estimating cell type proportions

**Figure 2:**
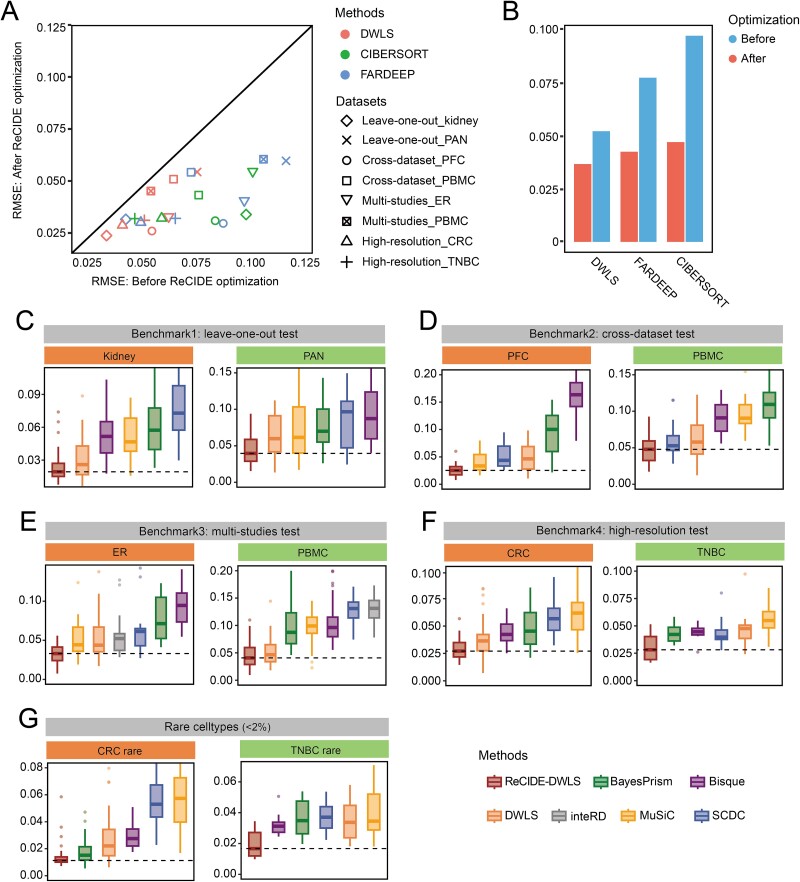
Benchmark performance of 11 deconvolution methods, including pre and post-ReCIDE optimization, across 4 scenarios. (a) Comparison of RMSE values before and after ReCIDE optimization for three single-reference-based methods (CIBERSORT, DWLS, and FARDEEP) in eight benchmark tests. (b) Average RMSE values for CIBERSORT, DWLS, and FARDEEP before and after ReCIDE optimization across eight benchmark tests. (c–f) Deconvolution accuracy assessed by RMSE values in leave-one-out, cross-dataset, multi-studies, and high-resolution scenarios, respectively. (g) Deconvolution accuracy based on RMSE values for rare cell types (constituting <2% of cells) in high-resolution tests.

The selection of the deconvolution kernel for ReCIDE involved an evaluation with three popular single-reference-based methods: CIBERSORT, DWLS, and FARDEEP. The assessment focused on gaging the enhancement in deconvolution accuracy relative to the standalone use of these methods. Remarkably, all three single-reference-based deconvolution methods, which traditionally do not consider cross-subject information within the reference, consistently exhibited improved performance when integrated into ReCIDE, as evidenced by the clearly reduced RMSE measure and improved PCC (Fig. 2a, Supplementary Fig. S1a). For example, using ReCIDE for optimization reduces the average of RMSE values from 0.0518 to 0.0366 and improves the average PCC from 0.812 to 0.877 for DWLS (Fig. 2b, Supplementary Fig. S1b). As DWLS consistently exhibited the highest accuracy among the three single-reference-based methods, both before and after ReCIDE optimization, it was chosen as the deconvolution kernel in ReCIDE, subsequently named ReCIDE–DWLS.

We then conducted a comparative analysis between ReCIDE–DWLS and five multi-reference–based methods (Bisque, BayesPrism, InteRD, MuSiC, and SCDC) across four benchmark test scenarios. Note that InteRD was exclusively examined in the third scenario. Based on the RMSE measure, ReCIDE–DWLS consistently secured the top rank in all eight benchmark tests across the four scenarios (Fig. 2c–f). The use of the PCC measure further corroborated that ReCIDE–DWLS exhibited the strongest correlation with the ground truth compared to all other methods (Supplementary Fig. S1c–f). Aside from ReCIDE–DWLS, no one method perform consistently better than the other methods based on both the RMSE and the PCC measures, and the ranks of these methods differ by using different measures.

The “high-resolution test” was designed to evaluate deconvolution performance on rare cell types (constituting <2% of cells). But the RMSE measure was strongly influenced by errors on major cell types. To address this, we recalculated the RMSE considering only rare cell types, revealing that ReCIDE maintained its top-ranking position among all methods. However, the rankings of other methods underwent changes, exemplified by BayesPrism, which initially ranked fourth but moved up to second in terms of RMSE for rare cell types in the CRC dataset under this scenario (Fig. 2g).

**Figure 3:**
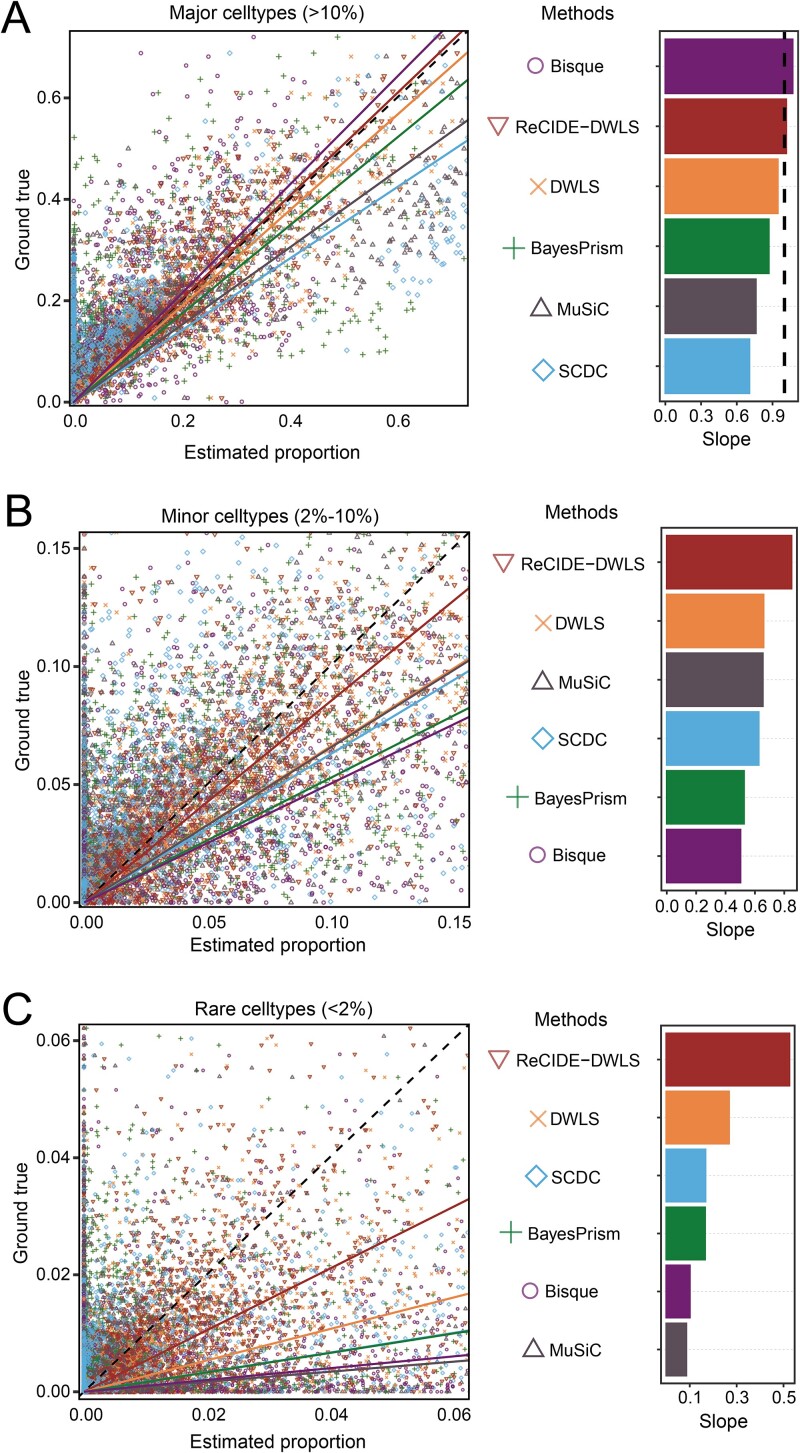
Fitted curves comparing estimated cell type proportions with true cell type proportions across various deconvolution methods. Panels (a–c) display fitted curves for major, minor, and rare cell types, respectively, generated by different deconvolution methods. The slopes of the fitted curves are depicted in the corresponding subfigures on the right.

To further inspect the performance of different methods on estimating the proportions of cell types with different sizes, we categorized cell types into three groups based on their sizes: major (constituting more than 10%), minor (more than 2% but less than 10%), and rare (constituting less than 2%). Then, for each method, we generated scatter plots of estimated versus true cell type proportions for all test subjects in the eight benchmark tests and calculated the slope of the linear regression fitting curve. A slope closer to 1 indicates more accurate estimation. The deconvolution accuracy of all methods showed a significant decrease from major to minor to rare cell types, reflecting the heightened challenges when cell types become rare **(**Fig. 3a–c**)**. Nevertheless, ReCIDE–DWLS emerged as the best method in each category, with its advantage over other methods notably increasing in scenarios involving minor and rare cell types. In rare cell types, the fitted line slope for ReCIDE–DWLS was 0.525, far outperforming other methods [the slopes range from 0.085 (MuSiC) to 0.267 (DWLS)], underscoring its exceptional performance in deconvolving rare cell types (Fig. 3c). This positions ReCIDE–DWLS as an appealing method for deconvoluting bulk data where rare cell types may hold particular clinical interest.

As such, ReCIDE exhibits a noteworthy enhancement in performance compared to current single-reference-based methods, and ReCIDE–DWLS demonstrates superiority over multi-reference-based methods, especially for estimating rare cell type proportions.

### Validating ReCIDE**–**DWLS’s performance using real bulk dataset

Rare cell types have been reported with clinical significance in various studies. In a scRNA-seq study on COVID-19, dendritic cells (DCs), constituting approximately 0.5%–2% in healthy individuals, were identified as significantly downregulated in COVID-19 patients compared to their healthy counterparts [44]. A recent scRNA-seq study on systemic lupus erythematosus (SLE) revealed a substantial downregulation of mucosal-associated invariant T cells (MAIT) in SLE patients in comparison to healthy individuals, and a significant upregulation of proliferative T and NK cells (Prolif) [25]. We therefore selected a bulk RNA-Seq dataset on COVID-19 (GSE231409), comprising 279 samples, and a microarray dataset on SLE (GSE50772) with 81 samples [32] as test datasets to evaluate the deconvolution performance on these specific cell types. The reference dataset for both tests was derived from PBMC scRNA-seq data of healthy individuals obtained from the COVID-19 study [24], comprising 23 healthy subjects. The comparative assessment included ReCIDE–DWLS, DWLS, BayesPrism, Bisque, MuSiC, and SCDC.

**Figure 4:**
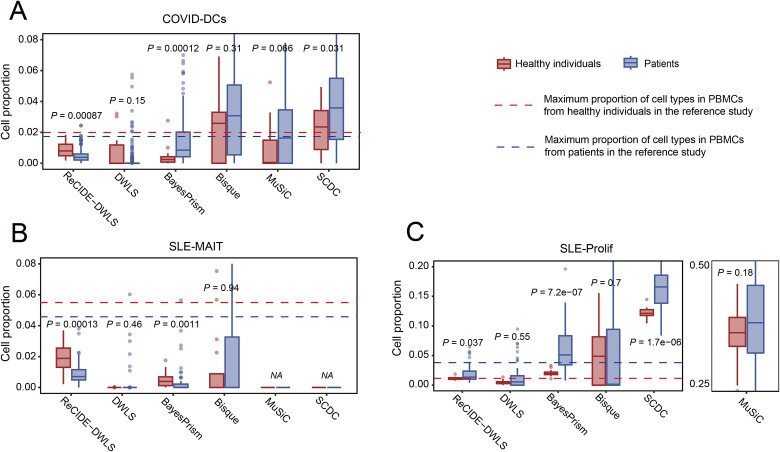
Deconvolution results of six methods applied to the COVID-19 and SLE datasets. (a) Deconvolution performance of six methods on DCs in the COVID-19 bulk RNA-seq dataset (0.5%–2% in healthy individuals and 0%–1.8% in patients). (b and c) Deconvolution performance of six methods on MAIT (0.015%–5.5% in healthy individuals and 0%–4.5% in patients) and Prolif (0%–1% in healthy individuals and 0%–4% in patients) cell types in the SLE microarray dataset.

In the bulk COVID-19 test, ReCIDE–DWLS yielded deconvolution results for the control group that aligned with the expected levels of DCs in healthy individuals (constituting approximately 0.5%–2%) and correctly identified the downregulation of DCs in COVID-19 patients (*P*-value = .00087; Fig. 4a), consistent with findings from the single-cell COVID-19 study [44]. All other deconvolution methods failed to correctly estimate the proportion of DCs cells in healthy individuals and failed to detect differences in the proportion of DCs between healthy individuals and COVID-19 patients. Additionally, BayesPrism and SCDC deconvolution results misidentify DCs cells proportion in patients as higher than in healthy individuals.

In the SLE microarray study, both ReCIDE–DWLS and BayesPrism estimated the levels of MAIT cells in the healthy individuals within the range reported by Perez et al. [25] and correctly identified the significant downregulation of MAIT cells in SLE patients (Fig. 4b). However, compared to BayesPrism, the deconvolution results of ReCIDE–DWLS show stronger significance between healthy individuals and SLE patients. On the other hand, in the deconvolution results of BayesPrism, a considerable proportion of SLE patients have an extremely low proportion of MAIT cells in their PBMCs (<1 × 10^−5^), which is inconsistent with reality [25]. The other methods failed to identify differences in the proportions of MAIT cells, and they underestimated the proportion of MAIT cells in control group. It is worth noting that Bisque, MuSiC, and SCDC estimated a considerable portion of the MAIT cells in both SLE patients and healthy individuals as 0. This might be attributed to significant batch effects between scRNA-seq data and microarray data, posing a challenge to all deconvolution methods. For Prolif cells, ReCIDE–DWLS not only successfully estimated the proportion of Prolif cells in both healthy individuals and SLE patients within their corresponding reported ranges but also correctly identified the significance of the difference in proportions (Fig. 4c). In contrast, although BayesPrism and SCDC correctly identified the significance of the differences, their estimations deviated from the true proportion ranges. Specifically, SCDC estimated the proportion of Prolif cells to be over 10% in healthy individuals and over 15% in SLE patients. Other methods failed to identify the significance of the difference, with MuSiC even overestimating the proportion of Prolif cells to be over 30%. Once again, in the testing based on SLE microarray data, ReCIDE–DWLS demonstrated superior performance in estimating rare cell type proportions.

### Application of ReCIDE–DWLS to bulk triple negative breast cancer datasets reveals prognostic relevance of subclasses of T and perivascular-like cells

Due to the superior performance of ReCIDE–DWLS in estimating cell type proportions, we applied this method to three bulk TNBC datasets: GSE164458 (482 patients, bulk RNA-seq), GSE58812 (107 patients, microarray), and the TCGA cohort (232 patients, bulk RNA-seq) [33, 34]. GSE164458 comprises RNA-seq from pre-treatment biopsies of patients undergoing neoadjuvant chemotherapy, in which information related to patient response in the face of chemotherapy is reported. GSE58812 and the TCGA cohort, on the other hand, consists of patients with reported survival outcomes. Our objective with ReCIDE–DWLS deconvolution was to identify clinically relevant cell types significantly associated with treatment response and patient prognosis. We utilized a reference dataset comprising scRNA-seq samples from four studies [28–30, 35, 45], totaling 39 patients (Supplementary Fig. S2**)**. To standardize cell type annotations across studies, we adopted annotations from Wu et al. [28], encompassing 49 cell types, and utilized SciBet to transfer these labels to the scRNA-seq data in the remaining three studies [26].

**Figure 5:**
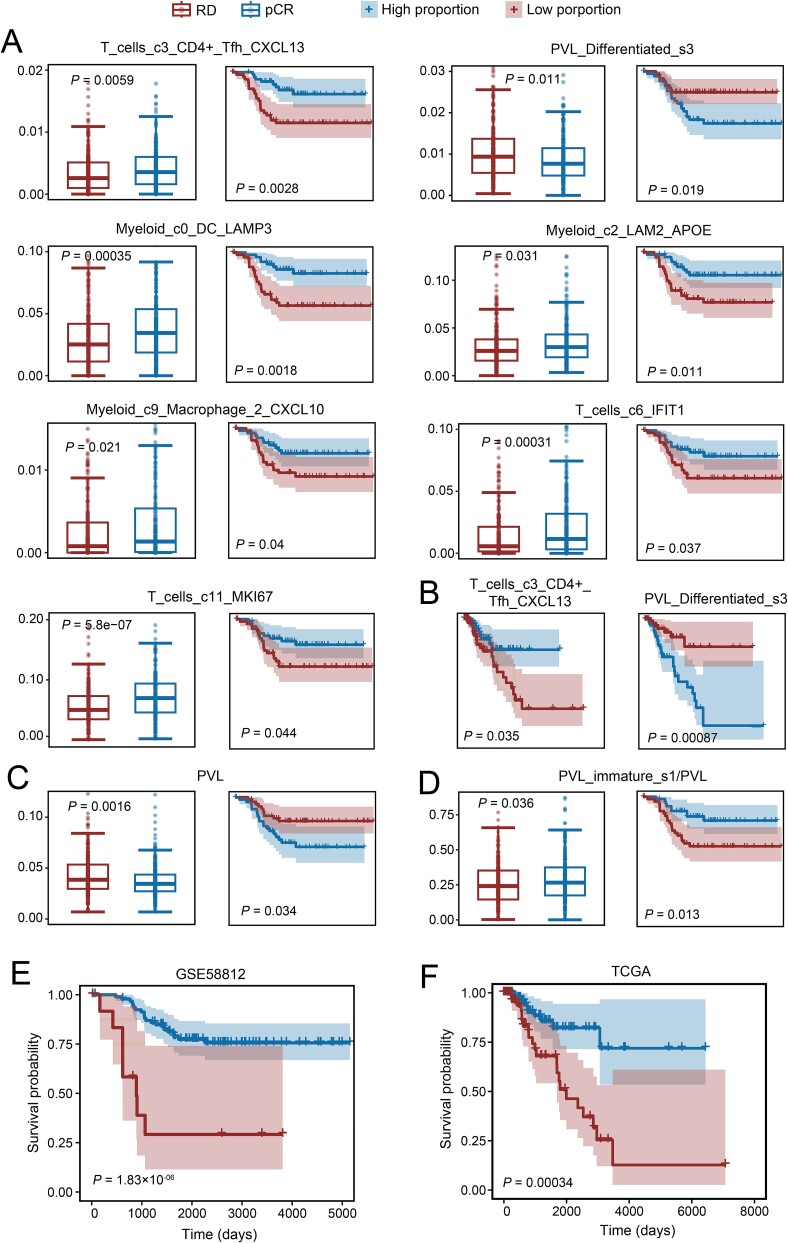
The clinical relevance of cell types deconvolved by ReCIDE–DWLS across three TNBC bulk datasets. (a) Seven cell types significantly associated with treatment response and prognosis (*P*-value <.05). For each cell type, boxplots compare proportions between RD (residual disease) and pCR (pathological complete response) patients in GSE164458, while Kaplan–Meier plots demonstrate survival time differences between patients with proportions greater or smaller than the median in GSE58812. (b) Cell types from (a) successfully validated in the TCGA dataset (*P*-value <.05), displaying prognostic curves of progression-free survival. (c) The correlation of PVL cell proportion with clinical response to therapy and prognosis in TNBC patients. (d) The correlation of PVL_immature_s1/PVL ratios with clinical response to therapy and prognosis in TNBC patients. (e) Kaplan–Meier plots derived from training on GSE58812. Prognostic scores for all patients in GSE58812 were calculated, and the score with the most significant *P*-value (.0125) was selected to plot the Kaplan–Meier plots (*P*-value = 1.83 × 10^−06^). (f) Kaplan–Meier plots resulting from applying the prognostic score threshold established from GSE58812 to the TCGA dataset (*P*-value = .00034).

Following the deconvolution of the three bulk datasets, we initially identified cell types whose proportions significantly correlate with treatment response in GSE164458 and patient prognosis in GSE58812, resulting in the identification of seven cell types (*P*-value <.05; Fig. 5a). Among these, six cell types (T_cells_c3_CD4 + _Tfh_CXCL13, T_cells_c6_IFIT1, T_cells_c11_MKI67, Myeloid_c0_DC_LAMP3, Myeloid_c2_LAM2_APOE, and Myeloid_c9_Macrophage_2_CXCL10) show positive associations with TNBC patient prognosis and treatment response, all belonging to immune cells. Additionally, a significant negative correlation exists between PVL_Differentiated_s3 and TNBC treatment response and prognosis, indicating that patients with lower proportions of PVL_Differentiated_s3 are more likely to have favorable treatment responses and prognosis. Subsequently, we validated these seven cell types using the TCGA cohort, successfully confirming the significance of two cell types, T_cells_c3_CD4 + _Tfh_CXCL13 and PVL_Differentiated_s3, both significantly correlated with TNBC patient prognosis (*P*-value in the TCGA dataset; Fig. 5b).

Our findings are corroborated by recent studies. For example, Zhang et al. demonstrated that TNBC patients with a higher proportion of CD4+ T cells expressing CXCL13 as marker genes (ranging from 0.02% to 7% in the scRNA-seq reference set) tend to benefit from anti-PD1 treatment [46]. Furthermore, elevated expression of the CXCL13 gene has been linked to improved prognosis in various cancers, including CRC and ovarian cancer [47, 48]. Wu et al. identified that PVL_Differentiated_s3 cells are strongly associated with immune evasion in multiple independent TNBC cohorts [49]. They categorized PVL cells into three subtypes: PVL_immature_s1, PVL_immature_s2, and PVL_Differentiated_s3 based on their differentiation states. Consequently, we further investigated the correlation between PVL and its subtypes with the prognosis of TNBC patients across three bulk datasets. Similar to PVL_Differentiated_s3, there is a significant negative correlation between PVL and TNBC treatment response and prognosis (Fig. 5c, Supplementary Fig. S6a). Interestingly, although no significant association was observed for PVL_immature_s1 alone, the ratios of PVL_immature_s1/PVL proportions—indicating the proportions of PVL_immature_s1 within PVL cells—were significantly positively correlated with treatment response and survival prognosis (Fig. 5d, Supplementary Fig. S6b). A recent study by DeFilippis et al. discovered that the immature-PVL marker CD36 is enriched in normal tissue regions and is associated with a favorable survival outcome in breast cancer [50], thus supporting our findings on PVL_immature_s1.

Given the associations of T_cells_c3_CD4 + _Tfh_CXCL13, PVL, PVL_immature_s1, and PVL_Differentiated_s3 cell types with TNBC patient prognosis, we sought to develop a prognostic assessment model for TNBC based on these cell types. Utilizing the aforementioned four cell types, we devised a prognostic score calculated as the product of the T_cells_c3_CD4 + _Tfh_CXCL13 to PVL_Differentiated_s3 ratio and the PVL_immature_s1 to PVL ratio, where a higher score suggests a more favorable clinical outcome. In the GSE58812 dataset, we computed the prognostic score for each patient and found that those with scores exceeding 0.0125 exhibited the most significantly improved prognosis compared to those below this threshold (*P*-value = 1.83 × 10^−06^; Fig. 5e), indicating its potential for patient stratification. We also computed prognostic scores of patients in the TCGA cohort and found that those with scores above the threshold (0.0125) had significantly longer survival times than those below (*P*-value = .00034; Fig. 5f), confirming the efficacy of our prognostic evaluation model. This underscores the capability of ReCIDE–DWLS in deciphering bulk expression profiles in TNBC patients for robust prognosis assessment.

## Discussion

In this study, we introduce ReCIDE which substantially enhances deconvolution accuracy by extending single-reference-based methods like CIBERSORT and DWLS to perform deconvolutions individually for each subject’s scRNA-seq data and consolidating the outcomes. Our extensive benchmark evaluation consistently demonstrates ReCIDE’s superiority over other methods, with ReCIDE–DWLS particularly excelling in estimating rare cell types, which holds substantial implications for uncovering clinically relevant low-abundance cell populations associated with diseases.

The presence of batch differences between bulk and single-cell reference data, stemming from both technical and biological reasons, poses a significant challenge for cell type deconvolution [11, 14]. While technical batch differences may be mitigated through more sophisticated algorithms or statistical frameworks [51], biological batch differences are inherent and challenging to overcome, especially for rare cell types. ReCIDE’s superior performance can be attributed to its more effective approach in addressing biological batch differences. Firstly, by processing the original reference into subject-specific references, diversity is introduced, reducing biological batch differences by increasing the likelihood of having references biologically relevant to the bulk data. Generally, ReCIDE’s performance benefits from a larger reference dataset, particularly when the initial number of subjects is limited, although the rate of improvement diminishes as the number of reference samples increases further (Supplementary Fig. S7). However, since predicting the precise number of reference samples needed for saturation is challenging, it is advisable to utilize as many reference samples as feasible. Secondly, optimizing the MGM to maximize the similarity between reference and bulk data further reduces biological batch differences. Without this optimization procedure, the RMSE values of ReCIDE–DWLS in the eight benchmark tests would increase by 0.6%–10.8% (Supplementary Fig. S8), demonstrating the positive impact of the optimization process. Thirdly, clustering of the deconvolution results enriches results closer to the ground truth. Despite significant variation in each estimation from the true proportions when using different reference sets, there is no consistent bias—sometimes overestimating and other times underestimating. Averaging these estimations therefore consistently improves the final estimate’s accuracy toward the true proportions. This approach is particularly effective for estimating rare cell type proportions, given that all existing methods perform poorly in this area. These simple yet effective treatments offer valuable directions for future development of deconvolution methods.

The comparison of ReCIDE–DWLS with other methods using real bulk datasets further underscores its efficacy. In both COVID-19 and SLE studies, ReCIDE–DWLS accurately estimates cell proportions for rare cell types, revealing biologically significant changes undetected by other methods. Its application to TNBC datasets uncovers the prognostic relevance of PVL cells, offering new insights into potential markers for patient outcomes. The ability to identify such associations demonstrates ReCIDE–DWLS’s strength in exploring clinically relevant cell types and its potential in large-scale clinical cohort studies.

Despite its strengths, ReCIDE has some limitations. This framework relies on a sufficient number of reference samples, but the optimal sample size required by ReCIDE varies in different scenarios. Determining the adequate number of samples needed in various contexts remains to be established. Additionally, multiple reference samples are required for deconvolution, necessitating high computational resources. In the COVID-19 dataset, which included 23 reference samples and 279 bulk samples, ReCIDE–DWLS completed the deconvolutions within 30 minutes using 50 CPUs running in parallel (Supplementary Table S1). Although it remains within an acceptable timeframe, further optimization is necessary to improve efficiency in the future.

Nonetheless, ReCIDE stands as a powerful framework for enhancing cell type deconvolution accuracy. ReCIDE–DWLS outperforms existing approaches in benchmark and real datasets, particularly excelling in estimating rare cell type proportions, positions it as a valuable tool in unraveling the complexities of cellular composition in large-scale clinical datasets, ultimately advancing our understanding of disease mechanisms and improving prognostic assessments.

**Key Points**

- ReCIDE introduces an innovative framework for robust estimation of cell type proportions by integrating single-reference-based deconvolutions.
- ReCIDE–DWLS outperforms existing approaches in benchmark and real datasets, particularly excelling in estimating rare cell type proportions.
- Application of ReCIDE–DWLS on TNBC datasets reveals correlation between subclasses of T cells and PVL cells, both of which are rare cell types, and the prognosis of TNBC patients, demonstrating its clinical relevance.

## Supplementary Material

Supplementary_information_0716_bbae422

Figures_bbae422

## Data Availability

All the datasets in this study are obtained from their public accessions. The detailed information including the accession codes and publication citations for all datasets can be seen in the Materials and Methods section.

## Code availability

Source codes for ReCIDE are freely available on github (https://github.com/TianLab-Bioinfo/ReCIDE).

## References

[1] Van de Sande B , Lee JS, Mutasa-Gottgens E. et al. Applications of single-cell RNA sequencing in drug discovery and development. *Nat Rev Drug Discov* 2023;22:496–520. 10.1038/s41573-023-00688-4. 37117846 PMC10141847

[2] Ye S , Li Q, Wu Y. et al. Integrative genomic and transcriptomic analysis reveals immune subtypes and prognostic markers in ovarian clear cell carcinoma. *Br J Cancer* 2022;126:1215–23. 10.1038/s41416-022-01705-w. 35043008 PMC9023449

[3] Clough E , Barrett T. The Gene Expression Omnibus database. *Methods Mol Biol* 2016;1418:93–110. 10.1007/978-1-4939-3578-9_5. 27008011 PMC4944384

[4] Hutter C , Zenklusen JC. The cancer genome atlas: creating lasting value beyond its data. *Cell* 2018;173:283–5. 10.1016/j.cell.2018.03.042. 29625045

[5] Wang R , Song S, Qin J. et al. Evolution of immune and stromal cell states and ecotypes during gastric adenocarcinoma progression. *Cancer Cell* 2023;41:1407–26.e9. 10.1016/j.ccell.2023.06.005. 37419119 PMC10528152

[6] Avila Cobos F , Vandesompele J, Mestdagh P. et al. Computational deconvolution of transcriptomics data from mixed cell populations. *Bioinformatics* 2018;34:1969–79. 10.1093/bioinformatics/bty019. 29351586

[7] Song L , Yang YT, Guo Q. et al. Cellular transcriptional alterations of peripheral blood in Alzheimer’s disease. *BMC Med* 2022;20:266. 10.1186/s12916-022-02472-4. 36031604 PMC9422129

[8] Newman AM , Liu CL, Green MR. et al. Robust enumeration of cell subsets from tissue expression profiles. *Nat Methods* 2015;12:453–7. 10.1038/nmeth.3337. 25822800 PMC4739640

[9] Hao Y , Yan M, Heath BR. et al. Fast and robust deconvolution of tumor infiltrating lymphocyte from expression profiles using least trimmed squares. *PLoS Comput Biol* 2019;15:e1006976. 10.1371/journal.pcbi.1006976. 31059559 PMC6522071

[10] Tsoucas D , Dong R, Chen H. et al. Accurate estimation of cell-type composition from gene expression data. *Nat Commun* 2019;10:2975. 10.1038/s41467-019-10802-z. 31278265 PMC6611906

[11] Wang X , Park J, Susztak K. et al. Bulk tissue cell type deconvolution with multi-subject single-cell expression reference. *Nat Commun* 2019;10:380. 10.1038/s41467-018-08023-x. 30670690 PMC6342984

[12] Jew B , Alvarez M, Rahmani E. et al. Accurate estimation of cell composition in bulk expression through robust integration of single-cell information. *Nat Commun* 2020;11:1971. 10.1038/s41467-020-15816-6. 32332754 PMC7181686

[13] Chu T , Wang Z, Pe’er D. et al. Cell type and gene expression deconvolution with BayesPrism enables Bayesian integrative analysis across bulk and single-cell RNA sequencing in oncology. *Nat Cancer* 2022;3:505–17. 10.1038/s43018-022-00356-3. 35469013 PMC9046084

[14] Dong M , Thennavan A, Urrutia E. et al. SCDC: bulk gene expression deconvolution by multiple single-cell RNA sequencing references. *Brief Bioinform* 2021;22:416–27. 10.1093/bib/bbz166. 31925417 PMC7820884

[15] Chen C , Leung YY, Ionita M. et al. Omnibus and robust deconvolution scheme for bulk RNA sequencing data integrating multiple single-cell reference sets and prior biological knowledge. *Bioinformatics* 2022;38:4530–6. 10.1093/bioinformatics/btac563. 35980155 PMC9525013

[16] Dai M , Pei X, Wang XJ. Accurate and fast cell marker gene identification with COSG. *Brief Bioinform* 2022;23:bbab579. 10.1093/bib/bbab579. 35048116

[17] Avila Cobos F , Alquicira-Hernandez J, Powell JE. et al. Benchmarking of cell type deconvolution pipelines for transcriptomics data. *Nat Commun* 2020;11:5650. 10.1038/s41467-020-19015-1. 33159064 PMC7648640

[18] Warnes GR , Bolker B, Lumley T. et al. Gmodels: various R programming tools for model fitting, 2022. https://CRAN.R-project.org/package=gmodels.

[19] Scrucca L , Fop M, Murphy TB. et al. Mclust 5: clustering, classification and density estimation using Gaussian finite mixture models. *R j* 2016;8:289–317. 10.32614/RJ-2016-021. 27818791 PMC5096736

[20] Fasolino M , Schwartz GW, Patil AR. et al. Single-cell multi-omics analysis of human pancreatic islets reveals novel cellular states in type 1 diabetes. *Nat Metab* 2022;4:284–99. 10.1038/s42255-022-00531-x. 35228745 PMC8938904

[21] Lake BB , Menon R, Winfree S. et al. An atlas of healthy and injured cell states and niches in the human kidney. *Nature* 2023;619:585–94. 10.1038/s41586-023-05769-3. 37468583 PMC10356613

[22] Xiong X , James BT, Boix CA. et al. Epigenomic dissection of Alzheimer’s disease pinpoints causal variants and reveals epigenome erosion. *Cell* 2023;186:4422–37.e21. 10.1016/j.cell.2023.08.040. 37774680 PMC10782612

[23] Morabito S , Miyoshi E, Michael N. et al. Single-nucleus chromatin accessibility and transcriptomic characterization of Alzheimer’s disease. *Nat Genet* 2021;53:1143–55. 10.1038/s41588-021-00894-z. 34239132 PMC8766217

[24] Stephenson E , Reynolds G, Botting RA. et al. Single-cell multi-omics analysis of the immune response in COVID-19. *Nat Med* 2021;27:904–16. 10.1038/s41591-021-01329-2. 33879890 PMC8121667

[25] Perez RK , Gordon MG, Subramaniam M. et al. Single-cell RNA-seq reveals cell type-specific molecular and genetic associations to lupus. *Science* 2022;376:eabf1970. 10.1126/science.abf1970. 35389781 PMC9297655

[26] Li C , Liu B, Kang B. et al. SciBet as a portable and fast single cell type identifier. *Nat Commun* 2020;11:1818. 10.1038/s41467-020-15523-2. 32286268 PMC7156687

[27] Hao Y , Hao S, Andersen-Nissen E. et al. Integrated analysis of multimodal single-cell data. *Cell* 2021;184:3573–87.e29. 10.1016/j.cell.2021.04.048. 34062119 PMC8238499

[28] Wu SZ , Al-Eryani G, Roden DL. et al. A single-cell and spatially resolved atlas of human breast cancers. *Nat Genet* 2021;53:1334–47. 10.1038/s41588-021-00911-1. 34493872 PMC9044823

[29] Pal B , Chen Y, Vaillant F. et al. A single-cell RNA expression atlas of normal, preneoplastic and tumorigenic states in the human breast. *EMBO J* 2021;40:e107333. 10.15252/embj.2020107333. 33950524 PMC8167363

[30] Bassez A , Vos H, Van Dyck L. et al. A single-cell map of intratumoral changes during anti-PD1 treatment of patients with breast cancer. *Nat Med* 2021;27:820–32. 10.1038/s41591-021-01323-8. 33958794

[31] Pelka K , Hofree M, Chen JH. et al. Spatially organized multicellular immune hubs in human colorectal cancer. *Cell* 2021;184:4734–52.e20. 10.1016/j.cell.2021.08.003. 34450029 PMC8772395

[32] Kennedy WP , Maciuca R, Wolslegel K. et al. Association of the interferon signature metric with serological disease manifestations but not global activity scores in multiple cohorts of patients with SLE. *Lupus Sci Med* 2015;2:e000080. 10.1136/lupus-2014-000080. 25861459 PMC4379884

[33] Jezequel P , Loussouarn D, Guerin-Charbonnel C. et al. Gene-expression molecular subtyping of triple-negative breast cancer tumours: importance of immune response. *Breast Cancer Res* 2015;17:43. 10.1186/s13058-015-0550-y. 25887482 PMC4389408

[34] Loibl S , O’Shaughnessy J, Untch M. et al. Addition of the PARP inhibitor veliparib plus carboplatin or carboplatin alone to standard neoadjuvant chemotherapy in triple-negative breast cancer (BrighTNess): a randomised, phase 3 trial. *Lancet Oncol* 2018;19:497–509. 10.1016/S1470-2045(18)30111-6. 29501363

[35] Xu K , Wang R, Chen Q. et al. Microenvironment components and spatially resolved single-cell transcriptome atlas of breast cancer metastatic axillary lymph nodes. *Acta Biochim Biophys Sin (Shanghai)* 2022;54:1336–48. 10.3724/abbs.2022131. 36148946 PMC9828062

[36] Hunt T. ModelMetrics: Rapid Calculation of Model Metrics, 2020. https://CRAN.R-project.org/package=ModelMetrics.

[37] Team RC . R: A Language and Environment for Statistical Computing, 2024. https://www.R-project.org/.

[38] McInnes L , Healy J, Melville J. UMAP: uniform manifold approximation and projection for dimension reduction. arXiv 2020;1802.03426.

[39] Becht E , McInnes L, Healy J. et al. Dimensionality reduction for visualizing single-cell data using UMAP. *Nat Biotechnol* 2019;37:38–44. 10.1038/nbt.4314. 30531897

[40] Kassambara A. Ggpubr: ‘ggplot2’ based publication ready plots, 2023. https://CRAN.R-project.org/package=ggpubr.

[41] Wickham H. ggplot2: Elegant Graphics for Data Analysis. Springer-Verlag, New York, 2016, 10.1007/978-3-319-24277-4.

[42] Kassambara A , Kosinski M, Biecek P. Survminer: drawing survival curves using ‘ggplot2’, 2021. https://CRAN.R-project.org/package=survminer.

[43] Tran KA , Addala V, Johnston RL. et al. Performance of tumour microenvironment deconvolution methods in breast cancer using single-cell simulated bulk mixtures. *Nat Commun* 2023;14:5758. 10.1038/s41467-023-41385-5. 37717006 PMC10505141

[44] Ren X , Wen W, Fan X. et al. COVID-19 immune features revealed by a large-scale single-cell transcriptome atlas. *Cell* 2021; 184:1895–913.e19. 10.1016/j.cell.2021.01.053. 33657410 PMC7857060

[45] Xu K , Wang R, Xie H. et al. Single-cell RNA sequencing reveals cell heterogeneity and transcriptome profile of breast cancer lymph node metastasis. *Oncogenesis* 2021;10:66. 10.1038/s41389-021-00355-6. 34611125 PMC8492772

[46] Zhang Y , Chen H, Mo H. et al. Single-cell analyses reveal key immune cell subsets associated with response to PD-L1 blockade in triple-negative breast cancer. *Cancer Cell* 2021;39:1578–93.e8. 10.1016/j.ccell.2021.09.010. 34653365

[47] Bindea G , Mlecnik B, Tosolini M. et al. Spatiotemporal dynamics of intratumoral immune cells reveal the immune landscape in human cancer. *Immunity* 2013;39:782–95. 10.1016/j.immuni.2013.10.003. 24138885

[48] Yang M , Lu J, Zhang G. et al. CXCL13 shapes immunoactive tumor microenvironment and enhances the efficacy of PD-1 checkpoint blockade in high-grade serous ovarian cancer. *J Immunother Cancer* 2021;9:e001136. 10.1136/jitc-2020-001136. 33452206 PMC7813306

[49] Wu SZ , Roden DL, Wang C. et al. Stromal cell diversity associated with immune evasion in human triple-negative breast cancer. *EMBO J* 2020;39:e104063. 10.15252/embj.2019104063. 32790115 PMC7527929

[50] DeFilippis RA , Chang H, Dumont N. et al. CD36 repression activates a multicellular stromal program shared by high mammographic density and tumor tissues. *Cancer Discov* 2012;2:826–39. 10.1158/2159-8290.CD-12-0107. 22777768 PMC3457705

[51] Newman AM , Steen CB, Liu CL. et al. Determining cell type abundance and expression from bulk tissues with digital cytometry. *Nat Biotechnol* 2019;37:773–82. 10.1038/s41587-019-0114-2. 31061481 PMC6610714

