# Supplementary material for: ReCIDE: robust estimation of cell type proportions by integrating single-reference-based deconvolutions
Source: Brief Bioinform. 2024 Aug 23;25(5):bbae422. doi: 10.1093/bib/bbae422 (PMC11342246; doi:10.1093/bib/bbae422)
Supplement: Supplementary_information_0716_bbae422 [file supplementary_information_0716_bbae422.docx]

**Supplementary Information**

**Supplementary Figures**

**Supplementary Figure S1**. Benchmark performance comparison of 11 deconvolution methods evaluated by Pearson correlation coefficient (PCC) values before and after ReCIDE optimization across four scenarios. (Related to **Figure 2**)

**Supplementary Figure S2**. Unified Manifold Approximation and Projection (UMAP) visualization depicting the integrated reference single-cell RNA sequencing (scRNA-Seq) atlas of Triple-Negative Breast Cancer (TNBC) patients. (Related to **Figure 5**)

**Supplementary Figure S3**. Comparison of cell type proportions estimated by ReCIDE-DWLS between RD (Residual Disease) and pCR (Pathological Complete Response) patients in the GSE164458 dataset for all cell types. (Related to **Figure 5A**)

**Supplementary Figure S4**. Kaplan-Meier plots based on estimated cell type proportions by ReCIDE-DWLS in the GSE58812 dataset for all cell types. (Related to **Figure 5A**)

**Supplementary Figure S5**. Kaplan-Meier plots based on estimated cell type proportions by ReCIDE-DWLS in the TCGA cohort for all cell types. (Related to **Figure 5B**)

**Supplementary Figure S6.** Kaplan-Meier plots depicting the relationship between PVL cell type proportions, PVL_immature_s1/PVL ratios, and the prognosis of TNBC patients in the TCGA cohort. (Related to **Figure 5C, D**.)

**Supplementary Figure S7**. The RMSE between the deconvolution results of ReCIDE-DWLS and the ground true varies with the number of reference samples.

**Supplementary Figure S8**. The RMSE between the deconvolution results of ReCIDE-DWLS and the ground truth in 8 benchmark tests, both before and after optimization.

**Supplementary Table**

**Supplementary Table S1**. The runtime of six deconvolution methods on the COVID-19 dataset.


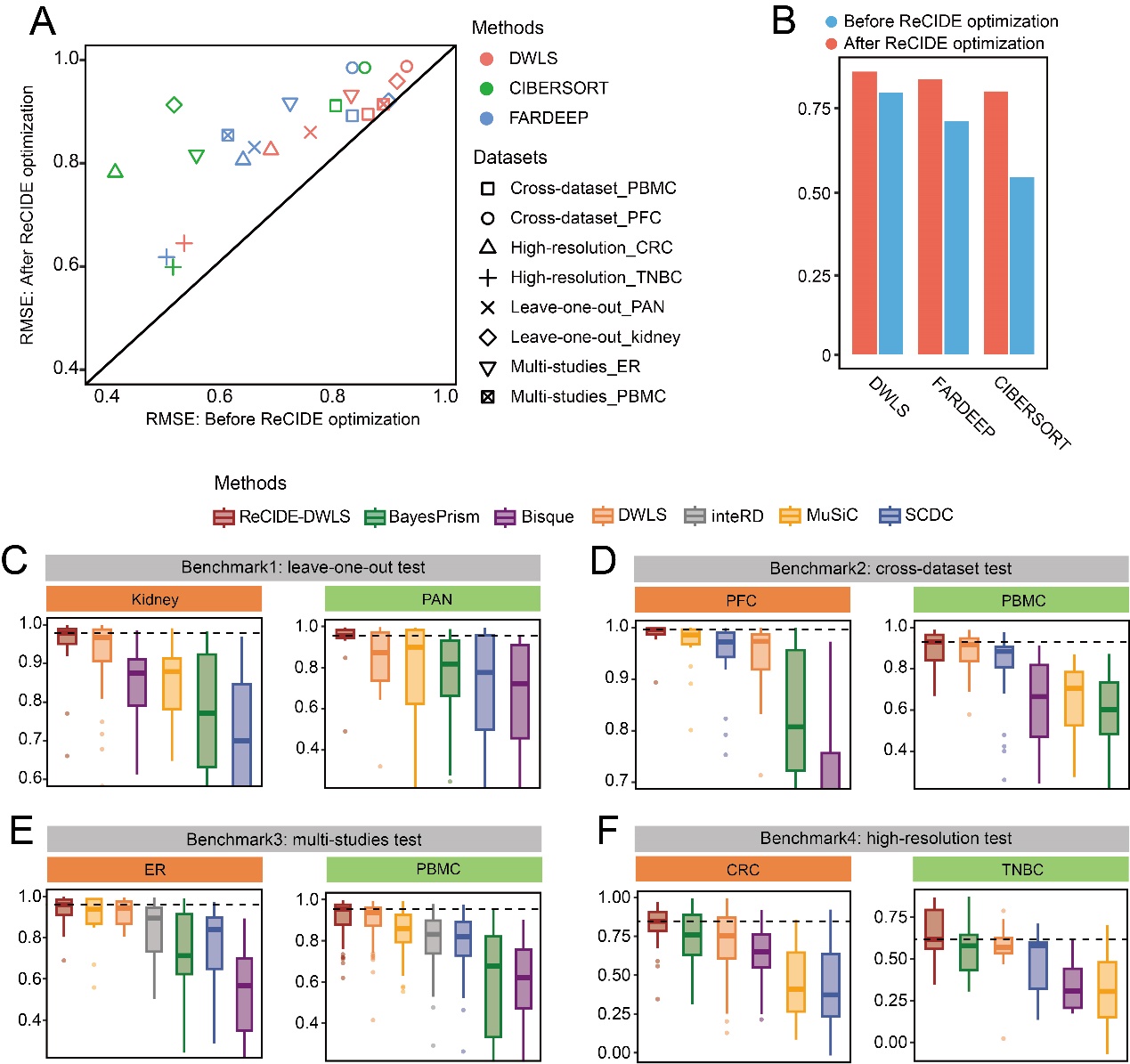


**Supplementary Figure S1**. Benchmark performance comparison of 11 deconvolution methods evaluated by Pearson correlation coefficient (PCC) values before and after ReCIDE optimization across four scenarios. Similar to Figure 2, except that the performance is assessed by PCC values. (Related to **Figure 2**)


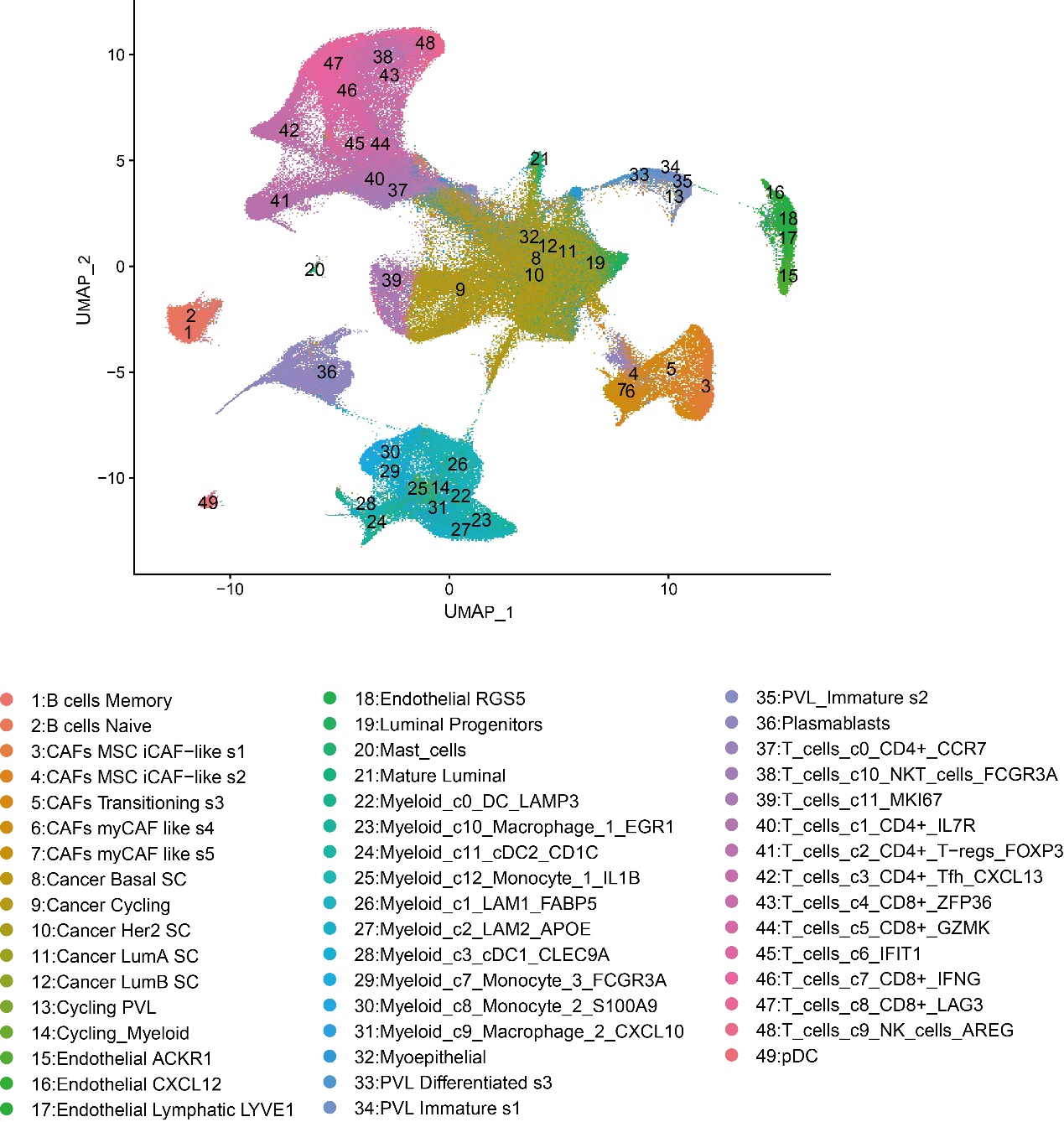


**Supplementary Figure S2**. Unified Manifold Approximation and Projection (UMAP) visualization depicting the integrated reference single-cell RNA sequencing (scRNA-Seq) atlas of Triple-Negative Breast Cancer (TNBC) patients. (Related to **Figure 5**)


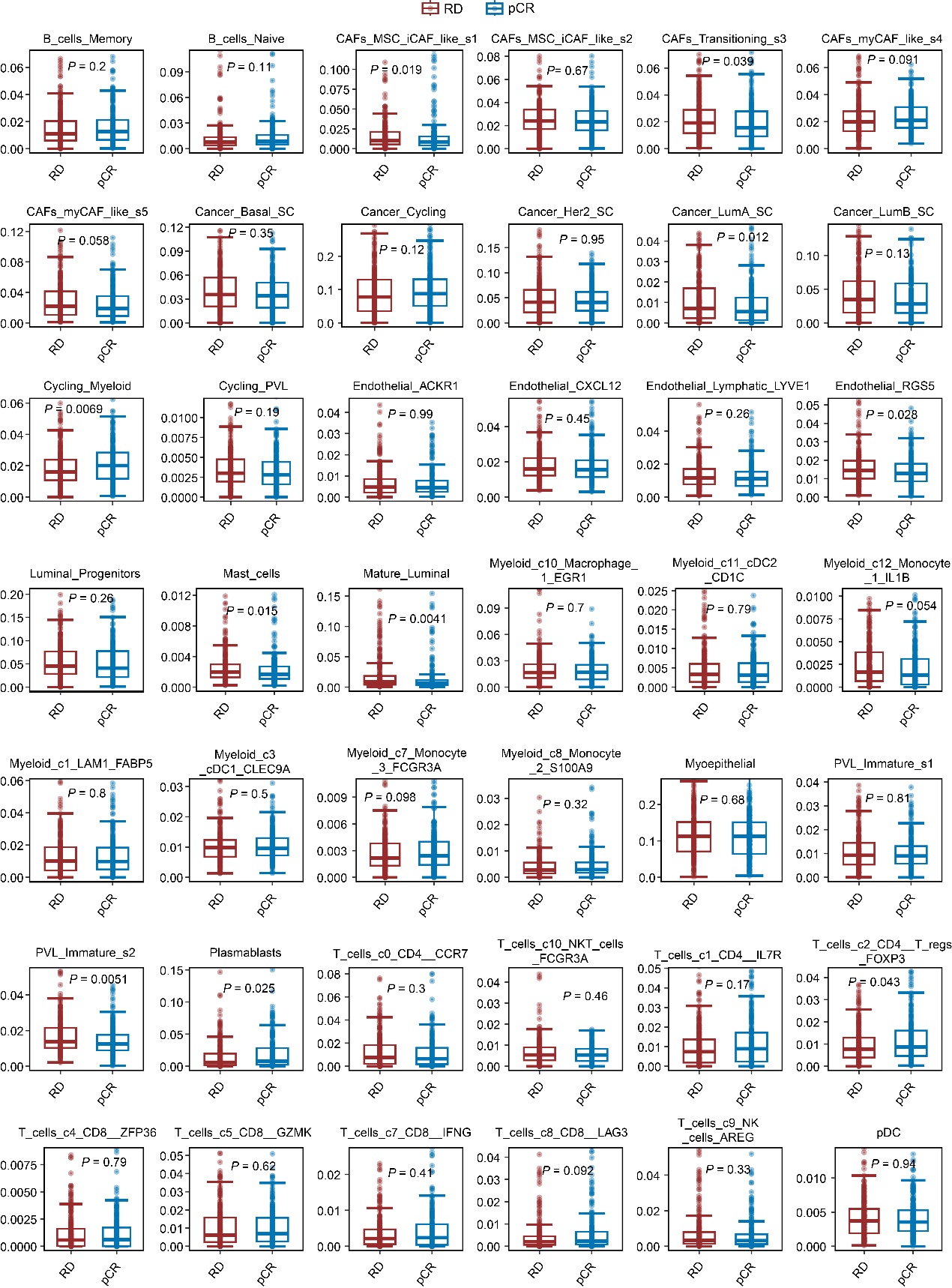


**Supplementary Figure S3**. Comparison of cell type proportions estimated by ReCIDE-DWLS between RD (Residual Disease) and pCR (Pathological Complete Response) patients in the GSE164458 dataset for all cell types. (Related to **Figure 5A**)


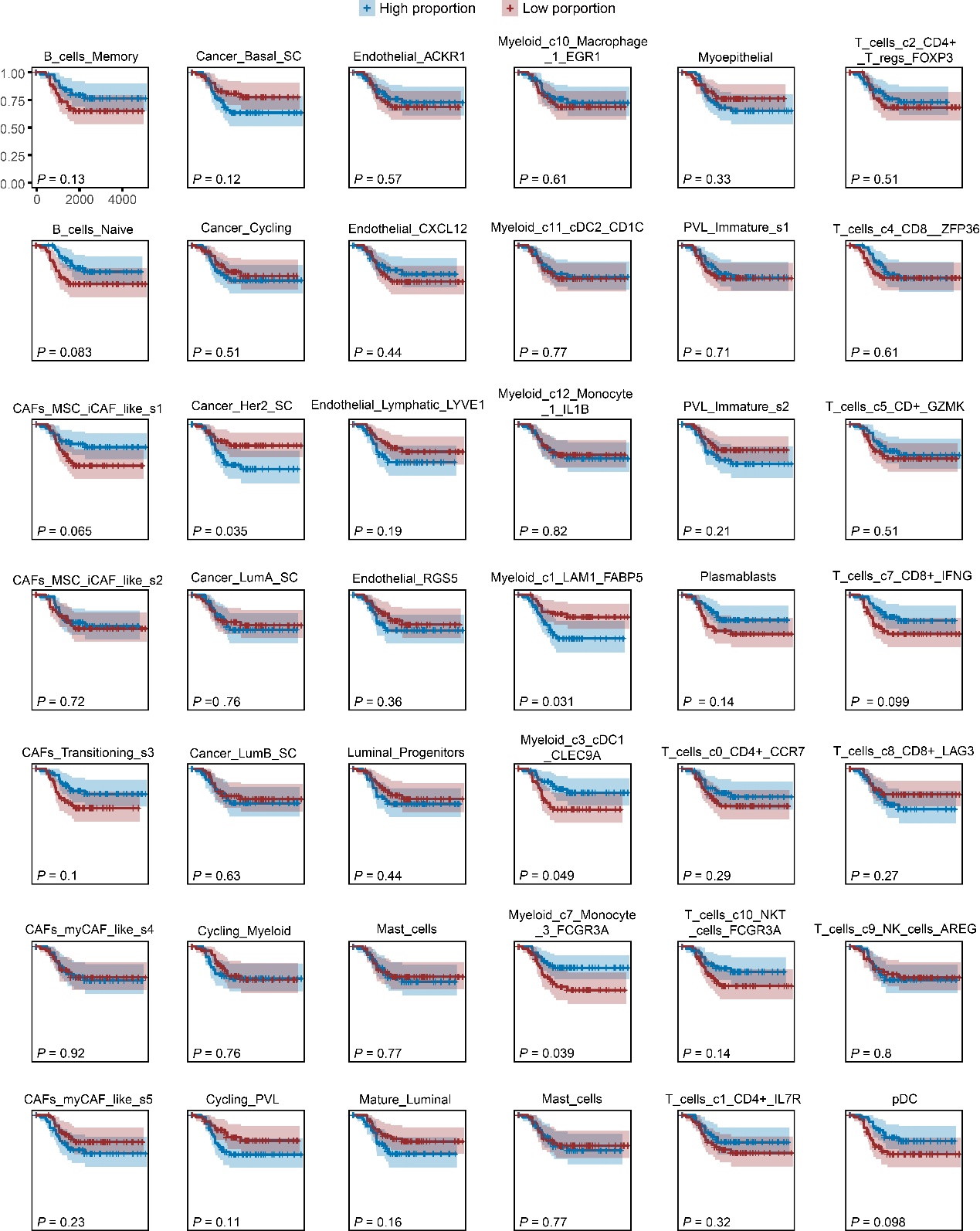


**Supplementary Figure S4**. Kaplan-Meier plots based on estimated cell type proportions by ReCIDE-DWLS in the GSE58812 dataset for all cell types. (Related to **Figure 5A**)


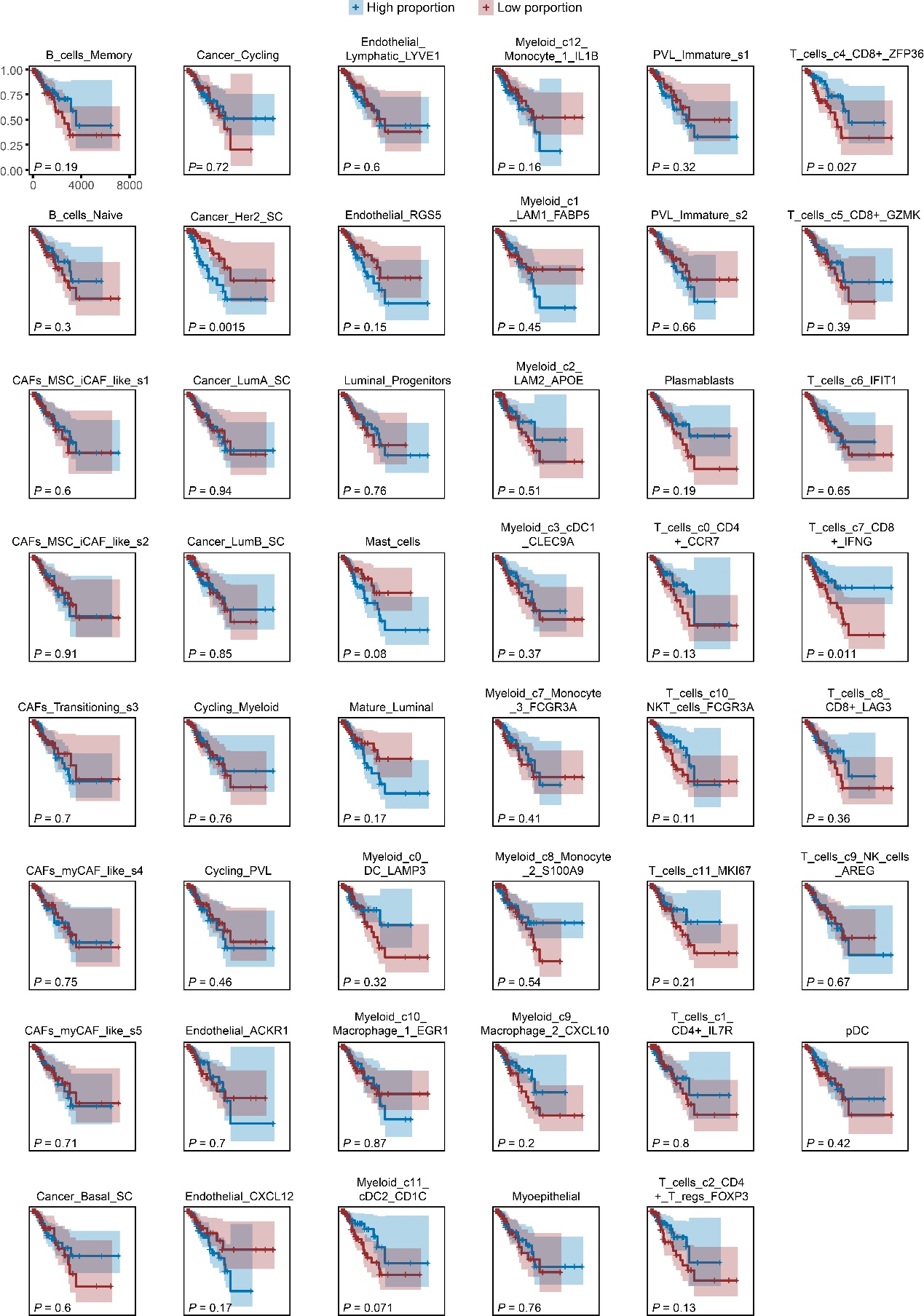


**Supplementary Figure S5**. Kaplan-Meier plots based on estimated cell type proportions by ReCIDE-DWLS in the TCGA cohort for all cell types. (Related to **Figure 5B**)


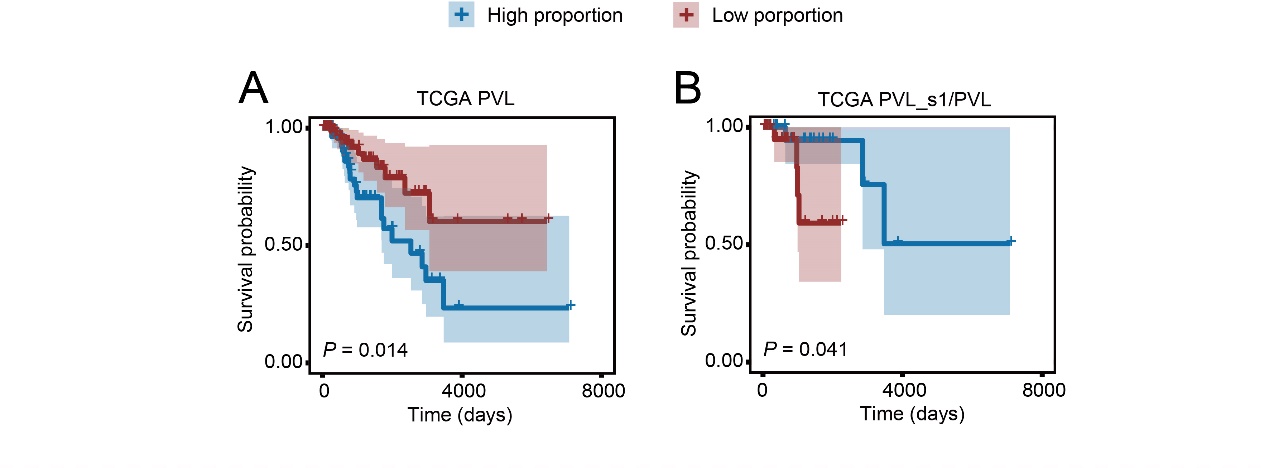


**Supplementary Figure S6**. Kaplan-Meier plots depicting the relationship between PVL cell type proportions (A), PVL_immature_s1/PVL ratios (B), and the prognosis of TNBC patients in the TCGA cohort. (Related to **Figure 5C, D**) Notably, for (B), Kaplan-Meier plots were generated using the top and bottom 15% of patients ranked by PVL_s1/PVL ratio, as dividing patients into two groups by the median did not yield a significant difference in prognosis.


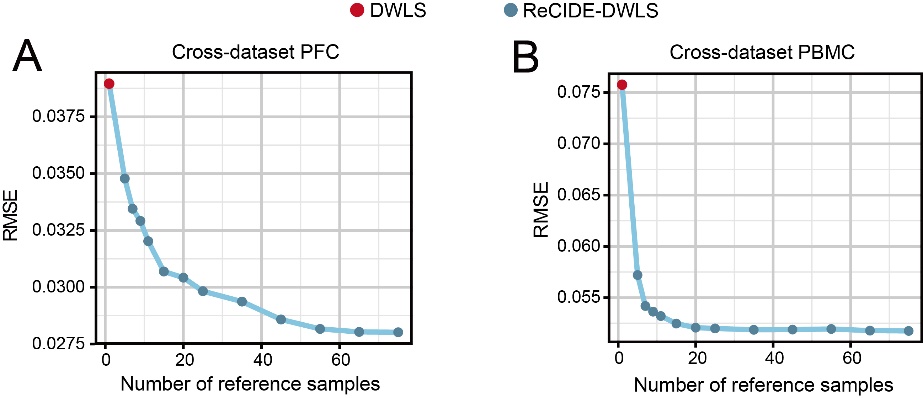


**Supplementary Figure S7**. The RMSE between the deconvolution results of ReCIDE-DWLS and the ground true varies with the number of reference samples. (A) Testing on the Cross-dataset PFC dataset. (B) Testing on the Cross-dataset PBMC dataset.


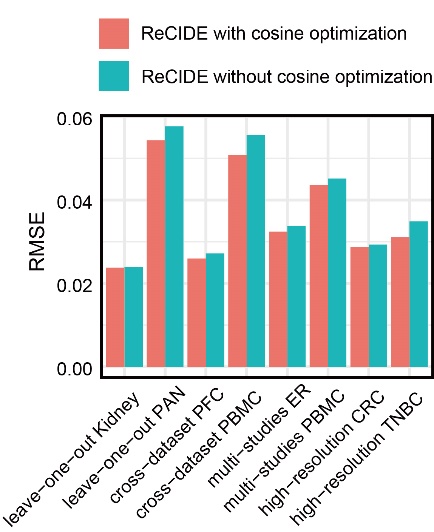


**Supplementary Figure S8**. The RMSE between the deconvolution results of ReCIDE-DWLS and the ground truth in 8 benchmark tests, both before and after optimization.

**Supplementary Table S1.** The runtime of six deconvolution methods on the COVID-19 dataset.

| Method | Time (min) |
| --- | --- |
| Bisque | 3.27 |
| Music | 3.56 |
| DWLS-COSG | 22.22 |
| ReCIDE-DWLS | 26.60 |
| CIBERSORT | 31.85 |
| BayesPrism | 35.21 |
